# Microeconomic Costs, Insurance, and Catastrophic Health Spending Among Patients With Acute Myocardial Infarction in India: Substudy of a Randomized Clinical Trial

**DOI:** 10.1001/jamanetworkopen.2019.3831

**Published:** 2019-05-17

**Authors:** Padinhare P. Mohanan, Mark D. Huffman, Abigail S. Baldridge, Raji Devarajan, Dimple Kondal, Lihui Zhao, Mumtaj Ali, Johny Joseph, Koshy Eapen, Mangalath N. Krishnan, Jaideep Menon, Manoj Thomas, Donald M. Lloyd-Jones, Sivadasanpillai Harikrishnan, Dorairaj Prabhakaran

**Affiliations:** 1 Department of Cardiology, WestFort Hi-Tech Hospital Ltd, Thrissur, India; 2 Northwestern University Feinberg School of Medicine, Chicago, Illinois; 3 Centre for Chronic Disease Control, Gurgaon, India; 4 Public Health Foundation of India, Gurgaon, India; 5 Department of Cardiology, Caritas Hospital, Kottyam, India; 6 Department of Cardiology, Samaritan Hospital, Pazhangad, India; 7 Department of Cardiology, Medical College Hospital, Kozikhode, India; 8 Department of Cardiology, Sree Narayana Institute of Medical Sciences, Ernakulam, India; 9 Department of Cardiology, St Joseph’s Hospital, Dharmagiri, India; 10 Department of Cardiology, Sree Chitra Tirunal Institute for Medical Science and Technology, Trivandrum, India; 11 London School of Hygiene and Tropical Medicine, London, United Kingdom

## Abstract

**Importance:**

Ischemic heart disease is the leading cause of death in India, and treatment can be costly.

**Objective:**

To evaluate individual- and household-level costs and impoverishing effects of acute myocardial infarction among patients in Kerala, India.

**Design, Setting, and Participants:**

This investigation was a prespecified substudy of the Acute Coronary Syndrome Quality Improvement in Kerala study, a stepped-wedge, cluster randomized clinical trial conducted between November 2014 and November 2016 across 63 hospitals in Kerala, India. In this cross-sectional substudy, individual- and household-level cost data were collected 30 days after hospital discharge from a sample of 2114 respondents from November 2014 to July 2016. Data were analyzed from July through October 2018 and in March 2019.

**Exposures:**

Health insurance status.

**Main Outcomes and Measures:**

The primary outcomes were detailed direct and indirect cost data associated with acute myocardial infarction and respondent ability to pay as well as catastrophic health spending and distress financing. Catastrophic health spending was defined as 40% or more of household expenditures minus food costs spent on health, and distress financing was defined as borrowing money or selling assets to cover health costs. Hierarchical regression models were used to evaluate the association between health insurance and measures of financial risk. Costs were converted from Indian rupees to international dollars (represented herein as “$”).

**Results:**

Among 2114 respondents, the mean (SD) age was 62.3 (12.7) years, 1521 (71.9%) were men, 1144 (54.1%) presented with an ST-segment elevation myocardial infarction, and 1600 (75.7%) had no health insurance. The median (interquartile range) expenditure among respondents was $480.4 ($112.5-$1733.0) per acute myocardial infarction encounter, largely driven by in-hospital expenditures. There was greater than 15-fold variability between the 25th and 75th percentiles. Individuals with or without health insurance had similar monthly incomes and annual household expenditures, yet individuals without health insurance had approximately $400 higher out-of-pocket cardiovascular health care costs (median [interquartile range] total cardiovascular expenditures among uninsured, $560.3 [$134.1-$1733.6] vs insured, $161.4 [$23.2-$1726.9]; *P* < .001). Individuals without health insurance also had a 24% higher risk of catastrophic health spending (adjusted risk ratio, 1.24; 95% CI, 1.07-1.43) and 3-fold higher risk of distress financing (adjusted risk ratio; 3.05; 95% CI, 1.45-6.44).

**Conclusions and Relevance:**

The results of this study indicate that acute myocardial infarction carries substantial financial risk for patients in Kerala. Expansion of health insurance may be an important strategy for financial risk protection to disrupt the poverty cycle associated with cardiovascular diseases in India.

## Key Points

**Question:**

What are the costs and risks of impoverishment for patients with acute myocardial infarction and their families in Kerala, India, and how do these factors vary by health insurance status?

**Findings:**

In this prespecified cross-sectional substudy of a randomized clinical trial, 2114 respondents reported $480 international dollars in out-of-pocket costs per acute myocardial infarction, with $400 international dollars in higher costs among individuals without insurance vs those with insurance. Catastrophic health spending was also higher among individuals without insurance (58.1% vs 39.9%) as was distress financing (9.7% vs 3.1%).

**Meaning:**

Survivors of acute myocardial infarction in Kerala may face high costs and risk for impoverishment, and expansion of insurance access and coverage should be considered for financial risk protection.

## Teaser

This prespecified cross-sectional substudy of a randomized clinical trial evaluates individual- and household-level costs and impoverishing factors related to acute myocardial infarction to assess whether acute myocardial infarction is associated with substantial financial risk for patients in Kerala, India.

## Introduction

Ischemic heart disease is the leading cause of death and disability in India,^1^ and treatment of acute and chronic manifestations of ischemic heart disease can be costly for patients and their families.^2^ Although the stepwise, inverse relationship between baseline socioeconomic position and incident cardiovascular disease (CVD) has been well described,^3^ including in Asia,^4^ the impoverishing effects of CVD have not been as widely reported. High-functioning health systems need to not only be accessible and responsive to patients’ needs but also provide financial risk protection for short- and long-term treatment. As a result, the United Nations Sustainable Development Goals includes target 3.8: “Achieve universal health coverage, including financial risk protection, access to quality essential health care services and access to safe, effective, quality, and affordable essential medicines and vaccines for all.”^5^

Individuals’ abilities to pay for health care expenditures are estimated by comparing out-of-pocket health care expenditures with household expenditures after accounting for subsistence, or food, expenditures. Catastrophic health spending occurs when individuals spend a high proportion of overall expenditures on health care with variable thresholds set, ranging from 10% to 40%,^6^ with subsequent poverty incidence rates ranging from 0.5% to 14%.^6^ Distress financing occurs when individuals try to buffer spending shocks by selling or mortgaging assets or borrowing money to cover health expenditures. A 2011 report from the south Indian state of Kerala (500 respondents) demonstrated a more than 60% prevalence rate of catastrophic health spending and distress financing among individuals with a recent acute myocardial infarction or stroke.^2^ India has subsequently enacted and scaled a social health insurance program, Rashtriya Swasthya Bima Yojana, to 36 million households to cover hospital expenses, with 2.1 million families covered in Kerala.^7^ However, this program does not seem to have reduced out-of-pocket spending.^8^

The Acute Coronary Syndrome Quality Improvement in Kerala (ACS QUIK) study was a stepped-wedge, cluster randomized clinical trial evaluating the effect of a quality improvement toolkit on clinical outcomes.^9,10^ As a prespecified substudy of ACS QUIK, the present study collected data on individual- and household-level expenditures associated with acute myocardial infarction. We sought to describe the individual and household costs associated with acute myocardial infarction; to assess how these costs compare with an individual’s ability to pay; to explore other patient-, hospital-, and treatment-level factors; and to evaluate the degree to which insurance mitigates risk for catastrophic health spending, distress financing, or both.

## Methods

### Overview of ACS QUIK

The methods and results of ACS QUIK (ClinicalTrials.gov identifier, NCT02256657) have been reported.^9,10^ In brief, 21 374 eligible participants with acute myocardial infarction were recruited from 63 hospitals in Kerala, India, from November 2014 to November 2016. The trial used a stepped-wedge cluster randomized design to evaluate the effect of a locally adapted quality improvement toolkit on the rate of 30-day major adverse events, defined as death, reinfarction as defined by the Third Universal Definition of Myocardial Infarction,^11^ stroke, and major bleeding as defined by the Global Utilization of Streptokinase and Tissue Plasminogen Activator for Occluded Coronary Arteries criteria.^12^ The unadjusted difference between the intervention and control groups (5.3% vs 6.4%) was no longer observed after adjustment for clustering and temporal trends (adjusted risk difference, −0.09% [95% CI, −1.32% to 1.14%]; adjusted odds ratio, 0.98 [95% CI, 0.80-1.21]). Rates of reperfusion and in-hospital and discharge medication were higher in the intervention group compared with the control group, which persisted after adjustment. The present report adheres to the Strengthening the Reporting of Observational Studies in Epidemiology (STROBE) reporting guideline for cross-sectional studies. The ACS QUIK trial and this substudy received institutional review board approval from local, national, and international bodies and was approved by the Indian Health Ministry Screening Committee. All participants or their delegates provided written informed consent.

### Sample Identification

We performed age-stratified (<50 years; 50-70 years; and >70 years) and sex-stratified random sampling of a subset of participants who presented for 30-day follow-up to evaluate microeconomic (ie, individual- and household-level) costs associated with acute myocardial infarction hospitalization. The initial recruitment target was 2200 respondents, which provided 95% power to detect differences in catastrophic health spending across age and sex strata, assuming a baseline catastrophic health spending prevalence rate of 60%.^2^ Recruitment for this substudy was planned to occur among sites and participants in the control period of the trial to minimize the potential influence of the toolkit intervention on costs, such as costs from higher rates of reperfusion and medications. However, slower-than-expected enrollment was identified during the first year of the trial owing to the length of the survey instrument and the time required from study personnel and participants. After consultation with the trial’s data and safety monitoring board, we revised our sampling frame in November 2015 to an unselected population within the overall trial to achieve our recruitment target by June 2016. Among all individuals who completed the microeconomic survey, we excluded 10 individuals who reported hospitalization outside the date range of the ACS QUIK trial (from November 2014 to November 2016). The most recent hospitalization was used for primary analysis to minimize recall bias. When hospitalizations were reported in the same month and year, we used the longer hospitalization.

### Assessment of Microeconomic Costs and Insurance Status

The instrument used to collect self-reported information on health care–associated expenditures, reimbursement, individual and household income, and household expenditures was developed in collaboration with experts from the World Bank and has been published.^2^ Health insurance was defined by self-report of having insurance at ACS QUIK admission or by any self-report of reimbursed expenditures from an insurer at the 30-day follow-up visit. Trained study personnel administered the survey in person to the participants, their delegates, or both during the 30-day follow-up research visit in the Malayalam or English language based on the participant’s preference. Costs were assessed in Indian rupees (Rs) and converted into 2015 international dollars based on purchasing power parities published by the World Bank (17.152 = international $)^13^; thus, unless otherwise indicated, all costs reported herein as “$” represent international dollars.

#### Cardiovascular Hospitalization and Total Health Care Expenditures

Inpatient cardiovascular hospitalization costs were calculated as the sum of self-reported spending on hospital admission, diagnostic tests, emergency department, food, treatment, ambulance, surgery, medicine, attendants, travel, and other self-reported inpatient costs. Total health care costs additionally included posthospitalization expenses on physicians, rehabilitation, home care, diagnostic tests, food, medicine, transportation, and other self-reported posthospitalization expenses. Out-of-pocket costs were calculated as the sum of all reported costs minus the total reimbursement by the insurance provider. A floor threshold of Rs 0 out-of-pocket costs was imposed where needed. Inpatient percentages were calculated only among patients with expenses reported.

#### Annual Household Expenses

Total annual household expenses were calculated as the sum of monthly expenses on rent, food, insurance, petrol, transportation, and educational expenses and annual expenses on goods, clothes, household fuel, health care, vehicle repair, property management, and any other self-reported household expenses. Nonfood expenses excluded monthly food expenses.

#### Proportionate Expenses

Cardiovascular disease expenditures as a proportion of annual household expenses were calculated as the out-of-pocket expenses on the most recent hospitalization as a proportion of the annual household expenditure. If Rs 0 total annual household expenditures were reported, then CVD expenses as a proportion of annual household expenditures were calculated as 100%.

### Catastrophic Health Spending and Distress Financing

Among patients who had out-of-pocket expenses and reported total nonfood annual household expenditures, catastrophic health spending was calculated. If out-of-pocket expenditures exceeded 40% of annual household expenditures minus food costs, then participants were considered to have experienced catastrophic health spending. Lower thresholds have been used to define catastrophic health spending (eg, 10% or 25%),^14^ but we reported results on the basis of our a priori conservative threshold that was based on previous reports.^15^ We also sought to compare prevalence rates in Kerala over time based on previous reports using the 40% threshold.^2^ Among patients who reported how health care costs were covered, participants were considered to have experienced distress financing if they reported covering their health care costs by borrowing money from friends, relatives, or a bank or they sold or mortgaged an asset.

### Statistical Analysis

We compared baseline demographic, clinical, and socioeconomic characteristics by insurance status using the *t* test for continuous, normally distributed covariates and the χ^2^ test for categorical covariates. We used the Wilcoxon rank sum test and the Kruskal-Wallis test for nonparametric analyses. We similarly compared CVD expenditures, annual household expenditures, changes in income, and rates of catastrophic health spending and distress financing by insurance status. Similar analyses were performed by sex, hospital type (ie, government, nonprofit/charity, or private hospital), and ST-segment elevation myocardial infarction (STEMI) or non-STEMI status.

To evaluate the association between insurance status and catastrophic health spending and distress financing, we created hierarchical multivariable binomial regression models. We adjusted for patient-level factors, including Global Registry of Acute Coronary Events risk score variables (ie, age, sex, STEMI or non-STEMI status, baseline systolic blood pressure, and heart rate),^16^ and baseline household income. We did not include serum creatinine owing to the high frequency of missing data. We did not include in-hospital heart failure, cardiogenic shock, or cardiac arrest in these models owing to collinearity and a small number of events (35 total). We further adjusted for hospital- and study-level factors by creating a random effect to adjust for hospital-level clustering and adjusting for ACS QUIK randomization group (intervention vs control groups). Finally, we sought to adjust for treatment-level factors, including reperfusion (eg, coronary artery bypass graft surgery, percutaneous coronary intervention, thrombolysis, or a combination thereof) and optimal in-hospital and discharge medications—defined as aspirin, second antiplatelet agent, anticoagulant (in-hospital only), β-blocker, and statin^17^—but the degree of missing data was too high. Complete case analysis was thus used for derivation of all variables and models. Data were analyzed from July through October 2018 and in March 2019. We used a 2-sided *P* < .05 to define statistical significance and Stata, version 15 (StataCorp), SAS, version 9.4 (SAS Institute Inc), and R, version 3.5.1 (R Foundation) to conduct statistical analyses. Code files used for generation of results can be downloaded from the internet.^18^

## Results

### Baseline Characteristics

The flowchart of 2114 participants is given in eFigure 1 in the Supplement. Participant characteristics by insurance status are reported in Table 1 and by sex in eTable 1 in the Supplement. Mean (SD) age of participants was 62.3 (12.7) years, 1521 (71.9%) were men, and 1144 (54.1%) presented with STEMI. There were modest differences between 1600 uninsured participants (75.7%) and 514 insured participants (24.3%) in terms of mean (SD) age (62.9 [12.9] years vs 60.3 [11.7] years, respectively; *P* < .001), STEMI prevalence rates (838 of 1600 [52.4%] vs 306 of 514 [59.5%]; *P* = .005), ACS QUIK intervention group status (875 of 1600 [54.7%] vs 210 of 514 [40.9%]; *P* < .001), and educational level (median [interquartile range {IQR}] years of education, 10 [6-12] years for uninsured vs 8 [5-10] years for insured, *P* < .001). Of 2114 participants overall, most were married (1751, 82.8%) and from a rural setting (1595, 75.5%). The median (IQR) time to survey completion following hospitalization was 31 (30-33) days in the uninsured group compared with 34 (30-49) days in the insured group (*P* < .001).

**Table 1.** Demographics, Clinical Presentation, and Laboratory Data From 2114 ACS QUIK Respondents Who Completed Microeconomic Questionnaires, Overall and by Insurance Status

Characteristic	Respondents, No.	Overall (N = 2114)	No Insurance (n = 1600)	Insurance (n = 514)	*P* Value^a^
Demographic at hospital presentation^b^					
Age, mean (SD), y	2114	62.3 (12.7)	62.9 (12.9)	60.3 (11.7)	<.001
Male, No. (%)	2114	1521 (71.9)	1162 (72.6)	359 (69.8)	.22
History of tobacco use, No. (%)	2114	618 (29.2)	456 (28.5)	162 (31.5)	.19
History of diabetes, No. (%)	2114	901 (42.6)	699 (43.7)	202 (39.3)	.08
Transferred, No. (%)	2114	907 (42.9)	709 (44.3)	198 (38.5)	.02
ST-segment elevation myocardial infarction, No. (%)	2114	1144 (54.1)	838 (52.4)	306 (59.5)	.005
Symptom-to-door time, median (IQR), min	2015	260 (120-960)	272 (120-980)	240 (130-879)	.82
Body weight, mean (SD), kg	2113	60.6 (10.8)	60.6 (11.1)	60.4 (9.7)	.74
Systolic blood pressure, mean (SD), mm Hg	2114	143.1 (30.6)	143.7 (30.5)	141.4 (30.9)	.15
Heart rate, mean (SD), bpm	2114	81.9 (19.7)	82.4 (20.2)	80.4 (18.0)	.04
Initial troponin, median (IQR), ng/mL	492	1.60 (0.48-6.09)	1.57 (0.47-5.70)	1.91 (0.69-8.71)	.16
LDL cholesterol, mean (SD), mg/dL	1587	121.0 (41.6)	120.7 (41.5)	122.1 (41.9)	.59
Triglycerides, median (IQR), mg/dL	1593	123 (93-174)	123 (95-175)	121 (90-173)	.51
Serum creatinine, median (IQR), mg/dL	980	1.1 (0.9-1.3)	1.1 (0.9-1.3)	1.0 (0.8-1.2)	.02
Fasting glucose, median (IQR), mg/dL	1394	130 (104-180)	133 (106-184)	120 (100-172)	.004
Hemoglobin, mean (SD), g/dL	2035	13.2 (2.1)	13.2 (2.1)	13.0 (2.1)	.15
ACS QUIK intervention, No. (%)	2114	1085 (51.3)	875 (54.7)	210 (40.9)	<.001
Socioeconomics, No. (%)^c^					
Married	2114	1751 (82.8)	1332 (83.3)	419 (81.5)	.36
Rural	2113	1595 (75.5)	1230 (76.9)	365 (71.2)	.009
Educational level, median (IQR), y	1941	9 (5-12)	10 (6-12)	8 (5-10)	<.001
Time to survey completion, median (IQR), d	1899	31 (30-34)	31 (30-33)	34 (30-49)	<.001
Unemployment, No. (%)	2114	824 (39.0)	584 (36.5)	240 (46.7)	<.001
Baseline monthly individual income, median (IQR)					
Rs	2074	9000 (2000-12 000)	10 000 (1500-12 000)	8000 (4000-10 000)	.56
International $^d^	2074	524.7 (116.6-699.6)	583.0 (87.5-699.6)	466.4 (233.2-583.0)	
Baseline monthly household income, median (IQR)					
Rs	2112	10 000 (8000-20 000)	15 000 (10 000-20 000)	7000 (4000-10 400)	<.001
International $^d^	2112	583.0 (466.4-1166.0)	874.5 (583.0-1166.0)	408.1 (233.2-606.3)	
Dependents, No. (%)					
<18 y	2114	1338 (63.3)	1056 (66.0)	282 (54.9)	<.001
>60 y	2114	1308 (61.9)	980 (61.3)	328 (63.8)	.30
Other individuals in household earning income, No. (%)	2114	1959 (92.7)	1494 (93.4)	465 (90.5)	.03

Abbreviations: ACS QUIK, Acute Coronary Syndrome Quality Improvement in Kerala; IQR, interquartile range; LDL, low-density lipoprotein; Rs, Indian rupees.

SI conversion factors: To convert troponin to micrograms per liter, multiple by 1.0; LDL cholesterol to millimoles per liter, by 0.0259; triglycerides to millimoles per liter, by 0.0113; serum creatinine to micromoles per liter, by 88.4; glucose to millimoles per liter, by 0.0555; and hemoglobin to grams per liter, by 10.0.

^a^ Unadjusted for differences.

^b^ Variables collected at hospital presentation among patients enrolled in the ACS QUIK study.

^c^ Variables collected from patients during follow-up at time of completion of the microeconomic assessment.

^d^ Uses 2015 conversion of 17.152 Indian rupees to equal 1 international dollar.^13^

Self-reported baseline monthly median (IQR) individual income was similar between uninsured and insured participants (uninsured, $583.0 [$87.5-$699.6] vs insured, $466.4 [$233.2-$583.0]; *P* = .56). By contrast, self-reported baseline monthly median (IQR) household income was higher among uninsured participants than insured participants ($874.5 [$583.0-$1166.0] vs $408.1 [$233.2-$606.3]; *P* < .001).

### Expenditures, Catastrophic Health Spending, and Distress Financing

Health care expenditures and household expenditures are reported by insurance status in Table 2. The median (IQR) expenditure among respondents was $480.4 ($112.5-$1733.0) per acute myocardial infarction encounter, largely driven by in-hospital expenditures. There was greater than 15-fold variability between the 25th and 75th percentiles. Total median (IQR) CVD-associated expenditures were higher among uninsured participants ($560.3 [$134.1-$1733.6]) compared with insured participants ($161.4 [$23.3-$1726.9]; *P* < .001), with an approximately 6-fold higher out-of-pocket, CVD-associated expenditures among individuals without insurance despite similar annual household expenditures. Catastrophic health spending was thus higher among individuals without insurance (533 of 1600 [58.1%] vs 111 of 514 [39.9%]; *P* < .001) as was distress financing (155 of 1600 [9.7%] vs 16 of 514 [3.1%]; *P* < .001). Expenditures are also reported by hospital type in eTable 2 in the Supplement and by STEMI status in eTable 3 in the Supplement. Costs are disaggregated as medians with corresponding IQRs for each cost subtype by STEMI vs non-STEMI status and hospital type in the Figure and by payment source in eFigure 2 in the Supplement.

**Table 2.** Expenditures and Incomes From 2114 ACS QUIK Respondents Who Completed Microeconomic Questionnaires

Expenditure	Respondents, No.	Overall (N = 2114)	No Insurance (n = 1600)	Insurance (n = 514)	*P* Value^a^
CVD expenditures, median (IQR), international $^b^^,^^c^					
Total CVD expense	2114	480.4 (112.5-1733.0)	560.3 (134.1-1733.6)	161.4 (23.3-1726.9)	<.001
Inpatient CVD expense	2113	356.8 (96.8-1623.7)	448.9 (116.6-1638.0)	110.2 (14.6-1587.3)	<.001
Out-of-pocket CVD expense	2114	374.3 (104.9-1316.5)	536.4 (131.7-1564.0)	87.9 (17.5-492.7)	<.001
Inpatient expense/total CVD expense, median (IQR), %^d^	2106	93.5 (80.5-99.0)	93.5 (82.6-98.8)	93.2 (70.4-100)	.26
Annual household expenditures, median (IQR), international $^c^^,^^e^					
Expense	1523	3847.9 (2512.8-5597.0)	3827.5 (2448.7-5879.8)	3847.9 (2938.4-4780.8)	.50
Nonsubsistence expense	1196	1539.2 (1107.7-2976.3)	1612.1 (1107.7-3157.1)	1399.3 (1049.4-2110.5)	.01
Out-of-pocket CVD expense/annual household expense, median (IQR), %^f^	1523	12.4 (4.1-44.6)	13.3 (4.3-54.4)	9.6 (3.1-20.9)	<.001
Income effects					
Any decrease in individual income, No. (%)	2113	964 (46.0)	634 (39.6)	330 (64.3)	<.001
Any decrease in household income, No. (%)	2113	927 (44.0)	610 (38.1)	317 (61.8)	<.001
Decrease in individual monthly income since hospitalization, median (IQR), international $^c^	2058	0.0 (0.0-174.9)	0.0 (0.0-174.9)	58.3 (0.0-174.9)	<.001
Decrease in household monthly income since hospitalization, median (IQR), international $^c^	2111	0.0 (0.0-174.9)	0.0 (0.0-174.9)	58.3 (0.0-174.9)	.006
Catastrophic and distress spending, No. (%)^g^					
Catastrophic health spending	1196	644 (54.0)	533 (58.1)	111 (39.9)	<.001
Distress financing	2114	171 (8.0)	155 (9.7)	16 (3.1)	<.001
Catastrophic health spending or distress financing	1311	772 (59.0)	648 (63.4)	124 (42.9)	<.001

Abbreviations: ACS QUIK, Acute Coronary Syndrome Quality Improvement in Kerala; CVD, cardiovascular disease; IQR, interquartile range.

^a^ Unadjusted for differences.

^b^ Total CVD expenses include inpatient expenses on hospital admission, tests, emergency department, food, treatment, ambulance, surgery, other self-reported expenses, medicine, attendants, and travel as well as posthospitalization expenses for physicians’ fees, rehabilitation, home care, other self-reported expenses, tests, food, medicines, and transportation. Out-of-pocket expense includes all CVD-related expenses minus any reimbursement for inpatient or posthospitalization services.

^c^ Uses 2015 conversion of 17.152 Indian rupees equal to 1 international dollar.^13^

^d^ Proportion is not calculated when total CVD expense is reported as zero or inpatient costs are missing.

^e^ Includes all expenses on food, gas, transportation, rent, insurance, educational level, goods, clothes, heating fuel, health care, vehicle repair, property management, and any other self-reported expenses. Nonsubsistence expenses exclude annual spending on food.

^f^ Proportion is not calculated when total household expense is missing.

^g^ Defined by out-of-pocket CVD expenses meeting or exceeding 40% of annual nonsubsistence household expenses. Distress financing is defined by borrowing from friends, relatives, or bank, or by selling an asset to cover CVD-related expenses.

**Figure.**
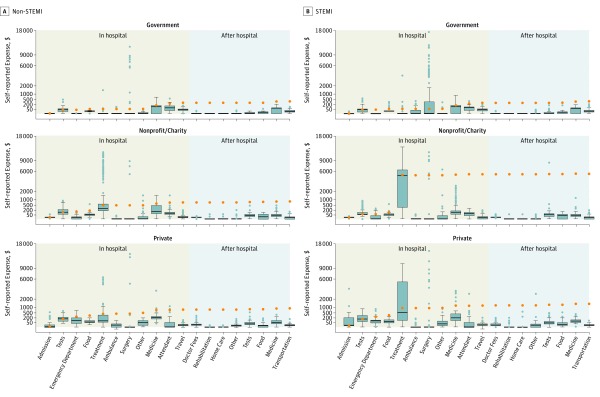
Cumulative Cardiovascular Disease Hospitalization Costs Costs are the sum of itemized medians for in-hospital and posthospitalization expenses among all 2114 Acute Coronary Syndrome Quality Improvement in Kerala trial respondents who completed microeconomic surveys, stratified by in-hospital and posthospital costs, ST-segment elevation myocardial infarction (STEMI) status, and hospital type. The top and bottom of the boxes indicate 25th and 75th percentiles; bands in boxes, medians; whiskers, 1.5 × (75th percentile − 50th percentile) and 1.5 × (25th percentile − 50th percentile); gray circles, outliers; orange circles, cumulative costs by cost category; and $, international dollars.

### Insurance, Catastrophic Health Spending, and Distress Financing

Table 3 reports the results of binomial regression models evaluating the associations between insurance and catastrophic health spending, distress financing, or both. In unadjusted analyses, lack of health insurance was associated with a 45% higher rate of catastrophic health spending (risk ratio [RR], 1.45; 95% CI, 1.25-1.70), which remained similar after adjustment for patient-level factors, including baseline income (adjusted RR, 1.49; 95% CI, 1.28-1.73), but was attenuated to 24% higher when adjusting for ACS QUIK intervention group and hospital cluster (adjusted RR, 1.24; 95% CI, 1.07-1.43). Similarly, lack of health insurance was associated with a 3-fold higher rate of distress financing (unadjusted RR, 3.11; 95% CI, 1.88-5.16), which was similarly attenuated after adjustment for patient-, study-, and hospital-level factors (adjusted RR, 3.05; 95% CI, 1.45-6.44).

**Table 3.** Association Between No Insurance on Catastrophic Health Spending, Distress Financing, or Both From 2114 ACS QUIK Respondents Who Completed Microeconomic Surveys

Model	Catastrophic Health Spending			Distress Financing			Catastrophic Health Spending or Distress Financing^a^		
	Respondents, No./Total No.	RR (95% CI)		Respondents, No./Total No.	RR (95% CI)		Respondents, No./Total No.	RR (95% CI)	
		No Cluster Effect	Study Effect^b^		No Cluster Effect	Study Effect		No Cluster Effect	Study Effect
Unadjusted^c^	644/1196	1.45 (1.25-1.70)	1.22 (1.03-1.43)	171/2114	3.11 (1.88-5.16)	2.80 (1.50-5.22)	772/1311	1.48 (1.28-1.70)	1.23 (1.06-1.43)
Adjusted									
Model 1^d^	644/1196	1.49 (1.28-1.74)	1.24 (1.07-1.43)	171/2114	3.14 (1.89-5.21)	2.87 (1.49-5.54)	772/1311	1.50 (1.31-1.73)	1.25 (1.09-1.44)
Model 2^e^	644/1196	1.49 (1.28-1.73)	ND^f^	170/2112	3.36 (1.99-5.67)	3.05 (1.45-6.44)	771/1310	1.51 (1.31-1.74)	1.23 (1.11-1.36)

Abbreviations: ACS QUIK, Acute Coronary Syndrome Quality Improvement in Kerala; ND, not determinable; RR, risk ratio.

^a^ Among 918 participants who were missing information to calculate catastrophic health spending, 115 provided a response for distress financing.

^b^ Study-level effects included ACS QUIK intervention and a random effect for hospital cluster.

^c^ Unadjusted model reference is insurance.

^d^ Adjusted model 1 is further adjusted for Global Registry of Acute Coronary Events risk score variables (age, sex, ST-segment elevation myocardial infarction or non–ST-segment elevation myocardial infarction status, systolic blood pressure, and heart rate). In-hospital heart failure, cardiogenic shock, and cardiac arrest were not included owing to collinearity and a small number of events.

^e^ Adjusted model 2 is further adjusted for baseline household income.

^f^ Binomial regression model did not converge.

## Discussion

### Summary

In this study of 2114 survivors of acute myocardial infarction in Kerala, 24% of whom had baseline health insurance, we report detailed individual- and household-level income and expenditure data, including expenditures associated with hospitalization and subsequent cardiovascular care. Median expenditures were less than $500 per episode, largely driven by in-hospital expenditures, but there was greater than 15-fold variability in expenditures between the 25th and 75th percentiles. We also quantified expected differences in expenditures by hospital type, with higher expenditures reported among respondents seeking care from nonprofit and private hospitals compared with public hospitals, which is concordant with previous reports from nationally representative samples.^19^ Individuals with or without health insurance had similar monthly incomes and similar annual household expenditures, yet individuals without baseline health insurance had approximately $400 higher out-of-pocket cardiovascular health care costs, a 45% greater risk of catastrophic health spending, and a 3-fold higher risk of distress financing.

### Results in Context

Previous facility-based studies evaluating the costs of acute cardiovascular care in India have reported higher CVD expenditures and higher rates of catastrophic health spending than the present study.^2,19,20^ These differences may be attributable to sampling frames (by location, disease states, or both), definitions, or temporal changes in income, expenditures, or insurance or combinations thereof. Furthermore, one previous study used a lower threshold to define catastrophic health spending (30% of nonsubsistence spending vs 40% in the present report),^20^ which also partially explains the lower prevalence rate observed in our study. Nevertheless, the rate of catastrophic health spending remains high, especially when compared with the incidence of catastrophic spending using global estimates aligned with the Sustainable Development Goals and other community-based reports of out-of-pocket spending from India.^21^ For example, using a health spending threshold of 10% of total consumption, global incidence was 9.7% in 2000 and rose to 11.7% in 2010,^14^ or approximately 5 times lower than in our study. However, distress financing was 3 to 6 times less common and lower than in previous reports, including in India,^2,19^ which may be driven by temporal increases in income, savings, or a combination thereof to better absorb health care–related cost shocks or greater wealth in Kerala compared with other states in India.^19^ Strategies to minimize the risk of “wealth shocks” will thus remain important not only to reduce the cycle of poverty from CVD but also to reduce associated mortality.^22^

Beyond broader initiatives to increase income and reduce health care–related expenditures, expanding access and enrollment in health insurance programs appears to be a central strategy to provide such financial risk protection, especially given the consistent association between insurance status and catastrophic health spending. A 2018 systematic review of 66 studies demonstrated that lack of health insurance was associated with a 2.7-fold higher risk of catastrophic health expenditures across a range of noncommunicable diseases, including CVD.^23^ Although health insurance should intuitively reduce out-of-pocket expenditures, not all studies show this association, especially among the lowest-income groups^24^ and through insurance obtained from private or nonprofit institutions.^14^ Incomplete insurance will not eliminate financial risk associated with health care and may counterintuitively increase financial risk when costs of treatment are not transparent. Within India, the effect of the central government’s Rashtriya Swasthya Bima Yojana health insurance program on catastrophic inpatient spending appears limited based on the 2004 to 2012 survey data from the National Sample Survey Organization, but a longer time horizon for programmatic implementation may be needed.^8^ State-level government health insurance programs to provide financial support to low-income households, such as the lottery-based Kerala Karunya Benevolent Fund, could complement national programs but have not yet been evaluated. Decreasing point-of-care fees for accessing health services and increasing access to credit or to disability insurance may be additional complementary strategies to reduce financial risk associated with short-term care for cardiovascular or other conditions that warrant further research.^6^ India’s March 2018 announcement of the Ayushman Bharat Pradhan Mantri Jan Arogya Yojana health insurance program, which would provide up to Rs 500 000 (approximately US $7000) in coverage annually to 100 million families (500 million individuals), would be a major investment in health that could substantially improve financial risk protection.^25^

### Strengths and Limitations

Our study has several strengths, including, to our knowledge, being the largest such study in India to report individual- and household-level detailed costs following hospitalization for an acute cardiovascular condition. Administrative claims data from government-sponsored programs, such as Rashtriya Swasthya Bima Yojana, have been shown to be insufficient for this level of granularity by condition or cost.^26^ Thus, we also used infrastructure from the ACS QUIK trial to capture these complex data using trained personnel. However, our study also has potential limitations. First, data on expenditures and costs were self-reported, which raises the risk of recall or social desirability bias. However, there is no central database on costs from which we could otherwise obtain these data, and other national reports relied on recall that was much further out from the period of hospitalization than our study. Further, we used a previously published instrument developed in collaboration with economists from the World Bank to collect detailed data to minimize these risks. Second, we changed our sampling frame during the study from an age- and sex-stratified random sampling frame to an unselected sampling frame given the low initial recruitment rates, which highlights the challenge of collecting these data. While the sample may not be representative of Kerala or of India, our study had sufficient power to demonstrate associations between patient-, hospital-, study-, and treatment-level factors with catastrophic health spending or distress financing. Third, we collected data from individuals who survived acute myocardial infarction, and these individuals may have different incomes, expenditures, and coping strategies than those who did not survive. Fourth, data were missing from some participants, including variables such as household expenditures, which were needed to estimate the prevalence of catastrophic health spending and may have influenced our results.

## Conclusions

The present results indicate that acute myocardial infarction carries substantial financial risk for patients in Kerala. Further expansion of health insurance access and coverage will be an important strategy for financial risk protection to disrupt the cycle of poverty from CVD in Kerala and in India.

## Notes

Supplement. **eTable 1.** Demographics, Clinical Presentation, and Laboratory Data From 2,114 ACS QUIK Respondents Who Completed Microeconomic Questionnaires, by Gender With Unadjusted *P* Value for Differences **eTable 2.** Detailed In-Hospital and Post-Hospitalization Expenditures (INT$) of 2,114 ACS QUIK Respondents Who Completed Microeconomic Questionnaires, Overall and by Hospital Type **eTable 3.** Detailed In-Hospital and Post-Hospitalization Expenditures (INT$) of 2,114 ACS QUIK Respondents Who Completed Microeconomic Questionnaires, Overall and by Myocardial Infarction Type **eFigure 1.** Flowchart of Participants Completing Microeconomic Survey During Steps Within the ACS QUIK Cluster Randomized Stepped Wedge Trial **eFigure 2.** Proportion of CVD Expenses Paid From Patient Reported Payment Sources by Insurance Status
