# Supplementary material for: Microeconomic Costs, Insurance, and Catastrophic Health Spending Among Patients With Acute Myocardial Infarction in India: Substudy of a Randomized Clinical Trial
Source: JAMA Netw Open. 2019 May 17;2(5):e193831. doi: 10.1001/jamanetworkopen.2019.3831 (PMC6537817; doi:10.1001/jamanetworkopen.2019.3831)
Supplement: Supplement. — eTable 1. Demographics, Clinical Presentation, and Laboratory Data From 2,114 ACS QUIK Respondents Who Completed Microeconomic Questionnaires, by Gender With Unadjusted P Value for Differences eTable 2. Detailed In-Hospital and Post-Hospitalization Expenditures (INT$) of 2,114 ACS QUIK Respondents Who Completed Microeconomic Questionnaires, Overall and by Hospital Type eTable 3. Detailed In-Hospital and Post-Hospitalization Expenditures (INT$) of 2,114 ACS QUIK Respondents Who Completed Microeconomic Questionnaires, Overall and by Myocardial Infarction Type eFigure 1. Flowchart of Participants Completing Microeconomic Survey During Steps Within the ACS QUIK Cluster Randomized Stepped Wedge Trial eFigure 2. Proportion of CVD Expenses Paid From Patient Reported Payment Sources by Insurance Status [file jamanetwopen-2-e193831-s001.pdf]

Supplementary Online Content

Mohanan PP, Huffman MD, Baldridge AS, et al; ACS QUIK Investigators. Microeconomic costs, insurance, and catastrophic health spending among patients with acute myocardial infarction in India: substudy of a randomized clinical trial. *JAMA Netw Open*. 2019;2(5):e193831. doi:10.1001/jamanetworkopen.2019.3831

eTable 1. Demographics, Clinical Presentation, and Laboratory Data From 2,114 ACS QUIK Respondents Who Completed Microeconomic Questionnaires, by Gender With Unadjusted *P* Value for Differences

eTable 2. Detailed In-Hospital and Post-Hospitalization Expenditures (INT$) of 2,114 ACS QUIK Respondents Who Completed Microeconomic Questionnaires, Overall and by Hospital Type

eTable 3. Detailed In-Hospital and Post-Hospitalization Expenditures (INT$) of 2,114 ACS QUIK Respondents Who Completed Microeconomic Questionnaires, Overall and by Myocardial Infarction Type

eFigure 1. Flowchart of Participants Completing Microeconomic Survey During Steps Within the ACS QUIK Cluster Randomized Stepped Wedge Trial

eFigure 2. Proportion of CVD Expenses Paid From Patient Reported Payment Sources by Insurance Status

This supplementary material has been provided by the authors to give readers additional information about their work.

eTable 1. Demographics, Clinical Presentation, and Laboratory Data From 2,114 ACS QUIK Respondents Who Completed Microeconomic Questionnaires, by Gender With Unadjusted *P* Value for Differences

Characteristic	N	Male n=1521	Female n=593	*P* value
**Demographics at Hospital Presentation**				
Age, mean (SD), years	2114	60.3 ± 12.3	67.4 ± 12.2	<.001
No insurance, n (%)	2114	1162 (76.4)	438 (73.9)	0.22
History of tobacco use, n (%)	2114	591 (38.9)	27 (4.6)	<.001
History of diabetes, n (%)	2114	595 (39.1)	306 (51.6)	<.001
Transferred, n (%)	2114	671 (44.1)	236 (39.8)	0.07
ST elevation myocardial infarction, n (%)	2114	889 (58.4)	255 (43.0)	<.001
Symptom-to-door time, median (IQR), min	2015	240 (120-893)	330 (140-1200)	<.001
Body weight, mean (SD), kg	2113	62.5 ± 10.3	55.5 ± 10.1	<.001
Systolic blood pressure, mean (SD), mmHg	2114	141.1 ± 30.2	148.5 ± 31.1	<.001
Heart rate, mean (SD), bpm	2114	80.1 ± 18.9	86.6 ± 20.8	<.001
Initial troponin, median (IQR), ng/ml	492	1.80 (0.57-7.32)	1.16 (0.33-3.10)	0.002
LDL cholesterol, mean (SD), mg/dl	1587	119.2 ± 40.5	125.5 ± 43.9	0.006
Triglycerides, median (IQR), mg/dl	1593	124 (93-176)	121 (95-164)	0.41
Serum creatinine, median (IQR), mg/dl	980	1.1 (0.9-1.3)	1.0 (0.8-1.2)	<.001
Fasting glucose, median (IQR), mg/dl	1394	128 (103-177)	135 (107-186)	0.10
Hemoglobin, mean (SD), mg/dl	2035	13.7 ± 1.9	11.8 ± 1.7	<.001
ACS QUIK Intervention n (%)	2114	793 (52.1)	292 (49.2)	0.23
**Socioeconomics**				
Married, n (%)	2114	1396 (91.8)	355 (59.9)	<.001
Rural, n (%)	2113	1129 (74.3)	466 (78.6)	0.04
Education level, median (IQR), years	1941	10 (6-12)	7 (4-10)	<.001
Time to survey completion, days (IQR)	1899	31 (30-34)	31 (30-34)	0.82
Unemployment, n (%)	2114	354 (23.3)	470 (79.3)	<.001
Baseline monthly individual income				
Median (IQR) Rs	2074	10000 (5000-15000)	2000 (0-10000)	<.001
Median (IQR) INT[a]	2074	583.0 (291.5-874.5)	116.6 (0.0-583.0)	
Baseline monthly household income				
Median (IQR) Rs	2112	10000 (8000-20000)	10000 (7000-18000)	0.008
Median (IQR) INT[a]	2112	583.0 (466.4-1166.0)	583.0 (408.1-1049.4)	

© 2019 Mohanan PP et al. *JAMA Network Open*.

Dependents < 18 years old, n (%)	2114	950 (62.5)	388 (65.4)	0.20
Dependents > 60 years old, n (%	2114	866 (56.9)	442 (74.5)	<.001
Other individuals in household earning income, n (%)	2114	1406 (92.4)	553 (93.3)	0.52

Abbreviations: LDL=low density lipoprotein, Rs=Indian rupees, INT=international dollar, IQR=interquartile range

© 2019 Mohanan PP et al. *JAMA Network Open*.

eTable 2. Detailed In-Hospital and Post-Hospitalization Expenditures (INT$) of 2,114 ACS QUIK Respondents Who Completed Microeconomic Questionnaires, Overall and by Hospital Type.

IQR=interquartile range, INT=international dollar

Expenditures	N	Overall n=2,114	Government Hospital n=456	Non-Profit/Charity Hospital n=1135	Private Hospital n=523	P-Value
**Median (IQR) In-Hospital Costs**						
Hospital Admission	1319	7.0 (0.0-7.0)	0.0 (0.0-0.0)	7.0 (7.0-7.0)	8.7 (1.2-87.5)	<0.001
Tests	1202	58.3 (43.7-174.9)	40.8 (7.0-58.3)	87.5 (58.3-174.9)	176.7 (87.5-288.3)	<0.001
Emergency Room	1299	0.0 (0.0-11.7)	0.0 (0.0-0.0)	0.0 (0.0-11.7)	104.9 (35.0-186.6)	<0.001
Food	934	58.3 (29.2-58.3)	17.5 (14.6-20.4)	58.3 (29.2-58.3)	81.6 (58.3-116.6)	<0.001
Treatment	1099	291.5 (58.3-4809.9)	0.0 (0.0-0.0)	583.0 (233.2-5830.2)	174.9 (78.7-1457.6)	<0.001
Ambulance	1048	0.0 (0.0-0.0)	0.0 (0.0-5.8)	0.0 (0.0-0.0)	0.0 (0.0-17.5)	<0.001
Surgery	1182	0.0 (0.0-0.0)	0.0 (0.0-0.0)	0.0 (0.0-0.0)	0.0 (0.0-0.0)	<0.001
Other	931	0.0 (0.0-29.2)	0.0 (0.0-0.0)	0.0 (0.0-29.2)	58.3 (11.7-116.6)	<0.001
Medicine	1224	116.6 (58.3-240.8)	116.6 (0.0-174.9)	116.6 (58.3-320.7)	240.8 (116.6-355.6)	<0.001
Attendant	2114	58.3 (26.2-116.6)	87.5 (26.2-116.6)	87.5 (29.2-116.6)	0.0 (0.0-58.3)	<0.001
Travel	2114	14.3 (5.8-29.2)	45.2 (17.5-58.3)	8.7 (0.0-17.5)	14.6 (8.7-23.3)	<0.001
Total In-Hospital Cost	917	1457.6 (583.0-7287.8)	44.7 (18.5-233.2)	5480.4 (874.5-7287.8)	1589.1 (878.3-6704.8)	<0.001
**Median (IQR) Post-Hospitalization Costs**						
Doctor Fees	1297	7.0 (0.0-11.7)	0.0 (0.0-0.0)	7.0 (7.0-8.7)	14.6 (11.7-29.2)	<0.001
Rehabilitation	1013	0.0 (0.0-0.0)	0.0 (0.0-0.0)	0.0 (0.0-0.0)	0.0 (0.0-0.0)	<0.001
Home Care	1132	0.0 (0.0-0.0)	0.0 (0.0-0.0)	0.0 (0.0-0.0)	0.0 (0.0-0.0)	<0.001
Other	909	0.0 (0.0-0.0)	0.0 (0.0-0.0)	0.0 (0.0-0.0)	8.7 (0.0-29.2)	<0.001
Tests	1150	17.5 (0.0-58.3)	0.0 (0.0-3.5)	29.2 (14.6-58.3)	42.0 (20.4-58.3)	<0.001
Food	832	5.8 (0.0-58.3)	2.9 (0.0-5.8)	14.6 (0.0-58.3)	8.7 (0.0-29.2)	<0.001
Medicine	1170	58.3 (14.6-70.0)	70.0 (0.0-87.5)	35.0 (17.5-58.3)	70.5 (37.9-116.6)	<0.001
Transport	1685	8.7 (2.9-17.5)	14.6 (5.8-29.2)	5.8 (0.0-11.7)	11.7 (5.8-17.5)	<0.001
Total Post-Hospitalization Cost	925	87.5 (20.4-174.9)	17.5 (8.7-87.5)	87.5 (35.0-174.9)	151.6 (98.5-291.5)	<0.001

© 2019 Mohanan PP et al. *JAMA Network Open*.

eTable 3. Detailed In-Hospital And Post-Hospitalization Expenditures (INT$) of 2,114 ACS QUIK Respondents Who Completed Microeconomic Questionnaires, Overall and by Myocardial Infarction Type.

IQR=interquartile range, INT=international dollar.

Expenditures	N	Overall n=2,114	NSTEMI n=970	STEMI n=1144	P-Value
**Median (IQR) In-Hospital Costs**					
Hospital Admission	1319	7.0 (0.0-7.0)	7.0 (1.2-7.0)	7.0 (0.0-7.0)	<.001
Tests	1202	58.3 (43.7-174.9)	87.5 (58.3-204.1)	58.3 (37.9-116.6)	<.001
Emergency Room	1299	0.0 (0.0-11.7)	0.1 (0.0-29.2)	0.0 (0.0-8.7)	<.001
Food	934	58.3 (29.2-58.3)	58.3 (29.2-58.3)	58.3 (26.2-58.3)	0.39
Treatment	1099	291.5 (58.3-4809.9)	233.2 (71.7-583.0)	524.7 (0.0-5830.2)	<.001
Ambulance	1048	0.0 (0.0-0.0)	0.0 (0.0-0.0)	0.0 (0.0-0.0)	0.51
Surgery	1182	0.0 (0.0-0.0)	0.0 (0.0-0.0)	0.0 (0.0-0.0)	<.001
Other	931	0.0 (0.0-29.2)	0.0 (0.0-58.3)	0.0 (0.0-29.2)	<.001
Medicine	1224	116.6 (58.3-240.8)	174.9 (58.3-320.7)	116.6 (58.3-201.4)	<.001
Attendant	2114	58.3 (26.2-116.6)	70.0 (23.3-116.6)	58.3 (29.2-116.6)	0.52
Travel	2114	14.3 (5.8-29.2)	11.7 (5.8-29.2)	14.6 (5.8-29.2)	0.30
Total In-Hospital Cost	917	1457.6 (583.0-7287.8)	932.8 (524.7-2798.5)	4955.7 (699.6-7287.8)	<.001
**Median (IQR) Post-Hospitalization Costs**					
Doctor Fees	1297	7.0 (0.0-11.7)	7.0 (0.0-11.7)	7.0 (0.0-8.7)	<.001
Rehabilitation	1013	0.0 (0.0-0.0)	0.0 (0.0-0.0)	0.0 (0.0-0.0)	0.10
Home Care	1132	0.0 (0.0-0.0)	0.0 (0.0-0.0)	0.0 (0.0-0.0)	0.05
Other	909	0.0 (0.0-0.0)	0.0 (0.0-5.8)	0.0 (0.0-0.0)	<.001
Tests	1150	17.5 (0.0-58.3)	17.5 (2.3-46.6)	14.6 (0.0-58.3)	0.35
Food	832	5.8 (0.0-58.3)	5.8 (0.0-29.2)	8.7 (0.0-58.3)	0.008
Medicine	1170	58.3 (14.6-70.0)	52.5 (17.5-70.0)	58.3 (11.7-75.8)	0.53
Transport	1685	8.7 (2.9-17.5)	8.7 (3.5-17.5)	8.7 (0.0-20.4)	0.99
Total Post-Hospitalization Cost	925	87.5 (20.4-174.9)	107.5 (35.0-174.9)	87.5 (15.2-174.9)	0.24

© 2019 Mohanan PP et al. *JAMA Network Open*.

eFigure 1. Flowchart of Participants Completing Microeconomic Survey During Steps Within the ACS QUIK Cluster Randomized Stepped Wedge Trial

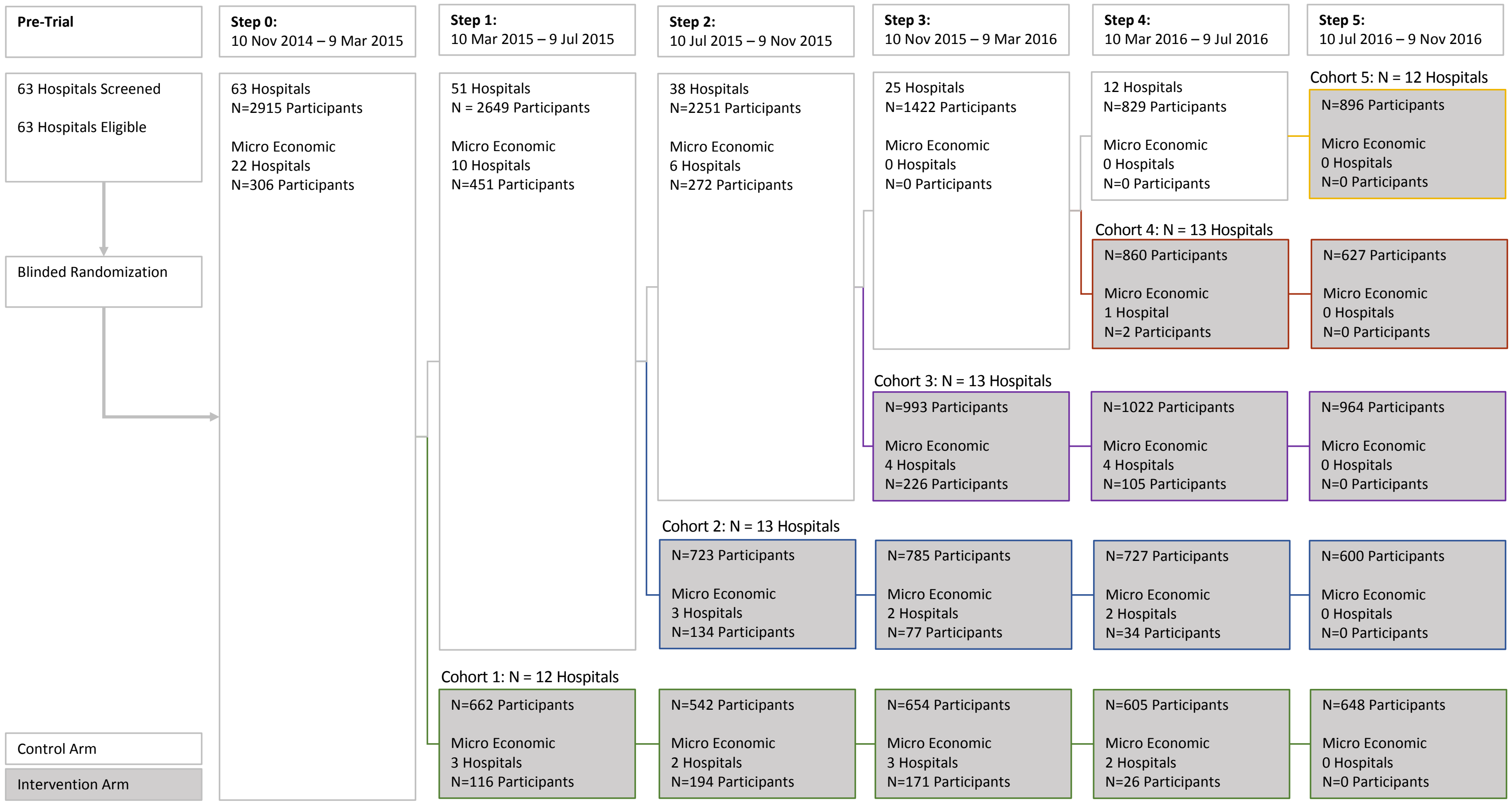

*Randomization occurred at a single point in time prior to patient enrollment. Hospitals were blinded to their assigned cohort until 2 weeks prior to the time point at which their cohort moved from the control to intervention stage.

© 2019 Mohanan PP et al. *JAMA Network Open*.

eFigure 2. Proportion of CVD Expenses Paid From Patient Reported Payment Sources by Insurance Status

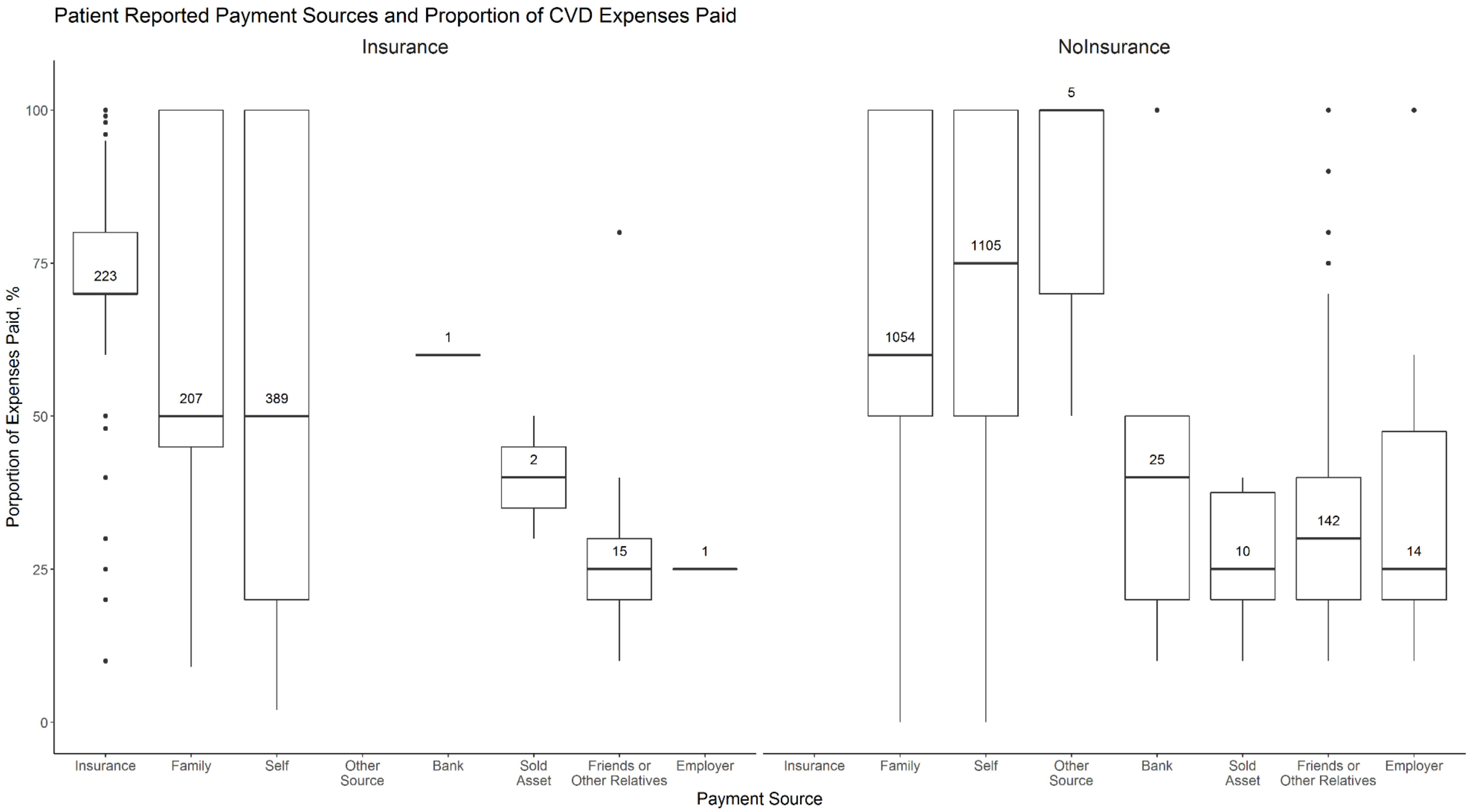

© 2019 Mohanan PP et al. *JAMA Network Open*.
